# Hyperactive Around the World? The History of ADHD in Global Perspective

**DOI:** 10.1093/shm/hkw127

**Published:** 2017-01-18

**Authors:** Matthew Smith

**Keywords:** ADHD; children; psychiatry; Ritalin; diagnosis; social constructivism; mental disorder

## Abstract

A recent study has claimed that the global rate of Attention Deficit Hyperactivity Disorder (ADHD) is 5.29%. Any variation in such rates in specific studies, argue the authors, was due to methodological problems, rather than differences in the actual distribution of ADHD. Such reports strengthen the flawed notion that ADHD is a universal and essential disorder, found in all human populations across time and place. While it is true that the concept of ADHD has spread from the USA, where it emerged during the late 1950s, to most corners of the globe, such superficial pronouncements mask profound differences in how ADHD has been interpreted in different countries and regions. In this paper, I compare ADHD's emergence in Canada, the UK, Scandinavia, China and India, arguing that, while ADHD can be considered a global phenomenon, behavioural and educational imperfections remain very much a product of local historical, cultural and political factors.

In May 2013, the fifth edition of the American Psychiatric Association’s *Diagnostic and Statistical Manual of Mental Disorders* (*DSM*) was published amidst a fog of controversy.^1^ Considered the American psychiatric bible, new editions of *DSM* have served as lenses through which the state of American psychiatry can be viewed. *DSM-II* (1968), for example, presented a profession dominated by psychoanalysis, but also in the midst of turmoil, as social psychiatrists and biological psychiatrists threatened to undermine psychoanalytic hegemony.^2^ In 1980, when *DSM-III* was published, it was clear that American psychiatry had entered a new biological—and pharmaceutical—age, in which mental illness was perceived in neurological terms and psychiatric treatment often started and finished with some scribbling on a prescription pad.^3^ Today, the furore surrounding the publication of *DSM-5* (yes, Arabic numerals have replaced Roman ones) appears to indicate a profession in flux once again. While psychiatry remains ensconced in a biological, psycho-pharmaceutical paradigm, many individuals, including many within psychiatry, have raised questions about the mechanistic determinism of neurologically-oriented psychiatry, the efficacy and safety of many psychiatric drugs, and the influence of the pharmaceutical industry on both diagnosis and treatment. As depicted in Steven Soderburgh’s *Side Effects* (2013, http://www.imdb.com/title/tt2053463/), American psychiatry has never been more ubiquitous, or more intrusive, but its role in American society is growing increasingly ambiguous.

But what about the role of American psychiatry in the global context? While American psychiatry may be in a muddle, American psychiatric disorders, with the invaluable help of *DSM*, have been on the march, spreading American notions of and pharmaceutical treatments for mental illness around the world like so many Happy Meals. As journalist Ethan Watters has argued, disorders which first took hold in the USA, ranging from anorexia to depression, are now commonplace in Asia and Africa.^4^ With these disorders come their pharmaceutical treatments. Psychiatrist P. Murali Doraiswamy has noted how, ‘as the Asian psyche becomes more Americanized, people from Bombay to Beijing are increasingly turning to pills for stress, insomnia, and depression’, rather than continuing the tradition in many Asian cultures of perceiving ‘suffering and sadness … as a part of spiritual growth and resilience’.^5^

Perhaps the best and most worrying example of such psychiatric colonisation is Attention Deficit Hyperactivity Disorder (ADHD), the subject of this essay, which has morphed from a disorder only recognised in the USA to one that is diagnosed and treated worldwide. ADHD has been the most commonly diagnosed childhood disorder in the USA since the 1960s. Children diagnosed with ADHD are perceived to be ‘imperfect’ by virtue of behaviours that are often recognised in childhood, including hyperactivity, inattention and impulsivity. The authors of the first description of what we would now call ADHD (which they called hyperkinetic impulse disorder) in 1957 noted that it was ‘striking’ that such behaviour was not so different from that of normal children.^6^ This quotidian aspect of ADHD behaviours in children helps to explain why diagnosis rates of the disorder have increased so much both in the USA and around the world. Unlike most ‘normal’ children, however, those diagnosed with ADHD are also typically prescribed psychiatric drugs to treat their symptoms, making them a particularly attractive market for pharmaceutical companies, who have turned from the reliable markets in North America to seek out ‘imperfect children’ elsewhere.

Great efforts have been made by proponents of ADHD to downplay the possibility that cultural, social and environmental factors play any role in the prevalence or diagnosis of the disorder. In 2007 an article by a team of largely Brazilian researchers calculated ADHD’s global prevalence. Attempting to explain the marked variations in ADHD rates in different countries, the team pored through epidemiological studies between 1978 and 2005, determining that the disorder occurred in 5.29 per cent of the world’s childhood population. Why the variable rates? These, the researchers argued, were due to the methodological differences that existed between studies, rather than differences in the actual distribution of ADHD internationally. In other words, children with ADHD were evenly distributed throughout the planet; no one country had a monopoly over the disorder.^7^ Underlying this explanation was the rather arrogant assumption that psychiatrists outside of where ADHD had been established were simply behind the times. Furthermore, the commentary which accompanied the article proclaimed that such findings confirmed the status of the disorder’s ‘identity as a bona fide mental disorder … as opposed to a social construction’, and weakened assertions that it was a ‘fraud propagated by the profit-dependent pharmaceutical industry and a high-status profession (psychiatry) looking for new roles’.^8^ This was despite the fact that the study itself was funded in part by pharmaceutical company Eli Lilly, which makes ADHD drug Strattera and for whom one of the authors worked as medical director. Two of the other authors were on the board of Eli Lilly and had ties to many other pharmaceutical companies, receiving funding from them and serving on their speakers’ bureaus. It was also notwithstanding ADHD’s contested status within Brazil, which has been neatly articulated by anthropologist Dominique Behague.^9^ Nevertheless, a similar article (funded this time by Johnson and Johnson, makers of the ADHD drug Concerta) contended that such global data undermined any idea that the disorder was ‘largely an American disorder’ rooted in ‘social and cultural factors that are most common in American society’.^10^

Leaving the methodological problems with such meta-analyses aside, along with evidence that, even within the USA, rates of ADHD vary considerably—ranging from 5.6 per cent in Nevada to 15.6 per cent in North Carolina—the magical 5.29 per cent figure amounts little more to than a grandiloquent distraction from the real story of ADHD’s propagation throughout the globe.^11^ For a start, the story is much more complicated, multi-faceted and nuanced than a simple tale of American psychiatric imperialism, or the ‘McDonaldization of childhood’.^12^ As medical history and anthropology is beginning to show, local differences are often the key to explaining and understanding variances in experiences of health and illness.^13^ Certainly, there is something rather insidious about the relentless global propagation of ADHD, which emerged in the USA not because psychiatrists suddenly realised that a certain percentage of American children were suffering from a mental disorder, but rather because of a host of political, ideological, demographic, technological, educational and environmental changes.^14^ But when ADHD’s history in different countries is examined more closely, a more complex story emerges.^15^ Instead of accepting the *DSM* version of ADHD unhesitatingly, psychiatrists—as well as parents and patients—from other countries or parts of different countries have adapted, modified and, indeed, contested American notions of ADHD. Just as the story of ADHD in the USA can only be properly understood by uncovering the historical context in which it emerged, the uptake of ADHD in other countries needs to be viewed in light of a host of social and cultural factors. In this paper, I analyse, using primarily medical literature, how ADHD has been taken up in Canada, the UK, Scandinavia, India and China, tracing the debates that have characterised its acceptance, rejection or modification in these jurisdictions. I have selected these countries for two primary reasons. First, they represent countries where the reception of ADHD has varied considerably and in fascinating ways, despite some of the countries’ similarities.^16^ Second, they are countries where ADHD and ADHD drugs, as well as other American psychiatric disorders and pharmacological treatments, have been aggressively marketed. My argument is that, while the notion that ADHD is a universal and essential disorder has been aggressively promoted by both psychiatrists and pharmaceutical companies, in practice, the picture has varied, with some countries embracing the disorder and others coming close to rejecting it.^17^ Underlying such variations have been an array of factors, ranging from more pluralistic approaches to psychiatry and the effect of highly influential figures in shaping opinion to different understandings of the meaning of childhood and what contributes to child behaviour. As with other aspects of ADHD and its history, the closer one examines the conditions in which it flourishes, the less it appears to be a universal, fixed glitch in neurological functioning, present in 5.29 per cent of the human population, and the more it becomes a product of culture. Imperfect children, when it comes to ADHD, are not born; they are constructed.

## Sleeping With an Elephant

During a 1969 speech, Canadian Prime Minister Pierre Trudeau (1919–2000) described how being a neighbour to the USA was ‘like sleeping with an elephant. No matter how friendly and even-tempered is the beast, if I can call it that, one is affected by every twitch and grunt’.^18^ The famous lines can be applied to many aspects of the Canadian–American relationship, but they have a certain ambiguity when it comes to medicine. On the one hand, the proximity of the ‘Darwinian US health care system’, characterised by private insurance and unequal access to services, has compelled Canadians to place a high value on their own public health care system.^19^ The medical profession in Canada, on the other hand, has long been influenced by American medicine. Not only are the medical education systems similar, with plenty of students crossing the border for training, but the 49th parallel is equally porous for qualified physicians. The most telling example of the elephantine effect American medicine has had on Canadian physicians is the Canadian Medical Association’s continual interest in adding elements of privatisation to the Canadian health care system, despite the popularity of Medicare.^20^ Given the contrast between a Canadian public that believes that free health care is indispensable and a Canadian medical profession that has often looked to the professional freedom and entrepreneurial spirit of their American colleagues with envy, the history of Canadian health care has long been characterised by the need to strike a balance between these disparate views. As Jacalyn Duffin and Lesley A. Falk have described, this search for balance can be traced to the very origins of Canadian health care.^21^ It is also characteristic of Canada’s approach to ADHD, particularly given the enormous influence of the USA.

The educational, ideological and geographical links between American and Canadian physicians, along with the cultural connections between the two countries more generally, contributed to ADHD’s relatively speedy migration across the 49th parallel. Whereas the first American medical articles discussing such a disorder, described as hyperkinetic impulse disorder, date from 1957, Canadian medical journals began to discuss it less than a decade later.^22^ A 1968 article in *Canadian Family Physician*, for example, discussed aggressive, impulsive, spontaneous and delinquent behaviour in adolescents, but not in terms of a distinct disorder.^23^ A year later, however, another article praised the use of stimulants in hyperactive children, claiming that they could ‘cause a rapid and amazing change, the child becoming calmer, much less active and amenable to teaching’.^24^ Soon the disorder was a popular topic in Canadian medical, educational and parenting publications.

What may surprise those who assume that ADHD is of purely American provenance is that many of the features that allowed it to become a globally-diagnosed condition that was treated with stimulants and could be identified in girls and adults (as well as just boys) were first recognised not by American researchers, but by a team working at McGill University in Montreal.^25^ Despite transforming ADHD in ways that allowed it to be diagnosed in epidemic numbers, however, the overall approach of the Montreal team to hyperactivity, and Canadian physicians more generally, tended to be more cautious, more holistic and more nuanced—or, in other words, more balanced—than that of their American neighbours.

In addition to their influential research on stimulant therapy, the Montreal group established of the first long-term follow-up study of ADHD.^26^ Comparing how hyperactive children fared academically, vocationally, cognitively, emotionally and socially with a control group, Gabrielle Weiss observed that, though both groups experienced difficulties, the ADHD group struggled more to cope with them. Moreover, these difficulties often persisted into adulthood and developed into more serious psychiatric problems. Such findings demonstrated that children with ADHD did not grow out of it, but rather continued to exhibit symptoms when they were adults, thus contributing to the emergence of adult ADHD in the 1990s. They also put pressure on educators and physicians to identify ADHD as a possible predictor for subsequent academic failure and mental illness. Despite her team’s initially positive findings regarding stimulants, however, Weiss admitted that drugs were not particularly effective in the long-term treatment of what had become a lifelong disorder. Indeed, she hoped that future treatment of hyperactive individuals would be more multidisciplinary, encompassing psychodynamic elements in particular.^27^ Weiss also acknowledged that while teachers often rated the hyperactive subjects worse that the control subjects, their later employers often found no difference, suggesting that the social environment, including a suitable vocation, was a key determinant of what types of behaviour were deemed to be problematic.^28^

Weiss’ intricate approach to understanding ADHD was also reflected in other members of her Montreal team, particularly psychiatrist Klaus Minde, whose longstanding interest in Africa and the cultural aspects of psychiatric disorders spurred him to investigate the disorder in Uganda.^29^ Like Weiss’ longitudinal research, Minde’s study was one of the first cross-cultural studies of the disorder to emerge during the 1970s, and the first to involve Africa. Although Minde and his co-author, Nancy Cohen, were surprised to find evidence of the disorder in Uganda, they also noted that, while Canadian children tended to be impulsive, their Ugandan counterparts tended to be more aggressive and short-tempered. Ugandan parents were also more likely to see such behaviour as a nuisance, rather than a medical disorder, and children with pronounced symptoms were simply not sent to school. Similarly, Minde’s comments in a 1975 editorial indicated that, while he believed hyperactivity was rooted in genetics, the social environment played an even larger role:

The majority of children who have difficulties with the world around them are not primarily hyperactive but are reacting to an environment that does not provide them with the necessary ingredients for their development. An understanding of these developmental needs can only be gained when we assess the total life-space of a child, which includes school, family and the child himself … . Children who receive little personal attention from their environment and get much of their entertainment from watching television are often bored and become restless, impulsive and aggressive. These children most often need people who they can trust, rather than drugs.^30^

Overlooking these social and environmental aspects, Minde warned, resulted in the over-prescription and over-dosage of drugs such as Ritalin.^31^

Another member of the Montreal team would challenge ideas about what lay behind the disruptive behaviour of children. Virginia Douglas had been involved in researching the effects stimulant drugs had on hyperactive children during the 1960s, but by the early 1970s she had also begun to wonder about the core symptoms of the disorder. What actually drove such energetic, impulsive and rumbunctious behaviour? Douglas suspected that attention-deficits were actually the underlying explanation.^32^ After conducting a series of trials, Douglas was not only convinced that inattention was the key feature in the hyperactivity of many children, but also that stimulant drugs could help such children improve their ability to focus and pay attention.^33^

The most obvious result of this shift in focus was that what had variously been described as hyperkinesis, hyperkinetic impulse disorder, minimal brain dysfunction (MBD) or simply hyperactivity, was now termed Attention Deficit Disorder (ADD) in *DSM-III* (1980), but there were other significant implications. The new focus on inattention meant that such children need not be troublemakers, delinquents or class clowns; they could also be day-dreamers who struggled academically but failed to cause enough disruption to be recognised as problematic, let alone pathological. Although hyperactivity would re-enter the terminology in *DSM-III-R* (1987), with the term Attention-Deficit/Hyperactivity Disorder (ADHD), the new emphasis on inattention still suggested that the disorder could go unnoticed for years.

The focus on inattention had a major impact for ADHD’s epidemiology. Specifically, it gave an answer to a conundrum that had been plaguing researchers for years, namely, why was the disorder seen more often in boys than girls?^34^ Now the answer appeared to be that girls were more often of the inattentive, rather than the hyperactive, type. Since they were less disruptive, they were less problematic in school and were subsequently under-diagnosed.^35^ The emphasis on attention also permitted more adults to be diagnosed, since they, too, tended to lack focus, rather than the ability to control their physical impulses. With Douglas’ stress on attention, ADD increasingly became perceived as a hidden disorder that was often overlooked and under-diagnosed.

Through the various contributions of the McGill team, it could be argued that Canadian understandings of ADHD shaped how Americans would see the disorder even more than the reverse. There were limits, however, to this influence. The relatively holistic and measured approach to hyperactivity espoused by psychiatrists such as Minde and Weiss remained more prominent in Canada than in the USA. Writing to the *Canadian Medical Association Journal* in 1988, Canadian physician Ray Holland described prescribing Ritalin to one of his hyperactive patients, but suspected food allergies in another, and found that a more structured social environment was most important for four others. For Holland, the *DSM* definition of the disorder had become too vague, leading to over-diagnosis and shifting the blame away from society itself, which was not set up properly for children to reach their potential.^36^ Similar letters, especially after the Canadian Paediatric Society came close to endorsing Ritalin in 1990, also indicate how Canadian physicians were more likely to resist conventional approaches to ADHD than their American counterparts.^37^ Even though Canadians contributed enormously to transforming ADHD into a global behemoth, not all Canadian physicians were happy with such developments

## Transatlantic Translations

When compared to their Canadian counterparts, British psychiatrists took somewhat longer to embrace ADHD, with hyperactivity first making an appearance in the *British Medical Journal* in 1968, when Philip Graham and Michael Rutter published one of their influential Isle of Wight surveys of child psychiatric disorders.^38^ Its first mention in the *Lancet* was in 1970, but this was merely a letter to the editor from some American psychiatrists, which was replied to by another American psychiatrist.^39^ Generally, most articles in British medical journals prior to the 1980s tended to discuss hyperactivity as a symptom of underlying conditions, ranging from encephalitis to phenylketonuria, rather than as a disorder unto itself.^40^ British media stories about hyperactivity were similarly rare, and those that did appear tended to depict the disorder as a North American, rather than a British, problem.^41^ The situation did not change until the 1990s, with one study calculating that while there were only 356 publications on the topic between 1985 and 1989, 6,158 had been written during the period 2005–2009.^42^

Although there were many reasons for the initial British reluctance, American ideas about the mental health of children had previously been influential in the UK. The American child guidance movement, for instance, found fertile soil in the UK following the First World War. As with ADHD in the USA during the 1950s and 1960s, child guidance in the UK was ‘an important constituent of a wider concern for political and social stability’, as John Stewart has described.^43^ But Stewart also cautions that the American medical model, dominated by psychiatrists, rather than psychologists, was not fully adopted in the UK.^44^ After the Second World War, British psychiatrists were less keen to embrace the biomedical model of mental illness than their colleagues across the Atlantic. Writing in 1975, criminologist Steven Box (1937–1987) argued that, while child psychiatry could be considered a ‘growth industry’ in the UK, there was ‘a strong anti-drug therapy feeling among British child psychiatric experts’.^45^ Put more bluntly by a consultant child psychiatrist in 1981: ‘I don’t practice chemical warfare against children’.^46^ What many British psychiatrists did maintain, long after their American cousins, was that childhood psychiatric disorders were often rooted in ‘psychosocial stresses’ which psychiatrists should attempt to ameliorate.^47^ When Rutter compared childhood mental illness on the Isle of Wight with inner London, for instance, he found that the London rates were twice as high. The reason for this was the additional stresses faced not only by children living in a chaotic metropolis, but also those their parents encountered.^48^ Another British study linked hyperactivity to maltreated children, with others stressing that ‘social and family factors may be the most important influences bearing on the behaviour of young children’ and downplaying neurological explanations.^49^ Or, as an editorial in the *Lancet* asked: ‘Are the Americans ahead of the British, or behind them, or do their children’s brains dysfunction in such an ostentatiously exotic transatlantic fashion that they require drug therapy?’^50^

The very terminology used to describe such troublesome children was also a factor. While British schoolchildren were often described by educators as ‘maladjusted’ or ‘medium educational subnormal’, British psychiatrists might diagnose them with ‘conduct disorder’, ‘school phobia’, ‘emotional disorder’ or even ‘autism’. Lest one believe that conduct disorder in the UK might equate to hyperactivity in the USA, British children with such problems were still seen in psychosocial rather than biological terms and drugs were rarely prescribed, highlighting fundamental conceptual differences.^51^ Even when British psychiatrists did discuss hyperactivity as a distinct disorder, the symptoms described were much more severe than those depicted in North American journals.^52^

Furthermore, in many such cases, environmental causes for this behaviour, such as food additives, heavy metal exposure and malnutrition, were often emphasised. Three of the British associations that raised awareness about the disorder during the 1970s, for example, stressed the role of environmental factors, including food chemicals, as important causes of hyperactivity.^53^ Such thinking was also reflected in how British psychiatrists responded to the food additive-free Feingold diet, designed by San Francisco allergist Ben Feingold (1899–1982) during the 1970s.^54^ When Feingold’s hypothesis re-emerged in the mid-2000s, not only were new trials conducted in the UK, but they also generated considerable media coverage and consumer attention.^55^

Nevertheless, there was little indication of a hyperactivity epidemic in Britain during the 1980s. A 1984 article stated that, while the disorder dominated 30–40 per cent of the clinical population seen by North American child psychiatrists, there had been only 73 hyperactive children seen at the Maudsley and Bethlem Royal Hospitals in London between 1968 and 1980.^56^ Attitudes, however, were changing. A *Lancet* editorial in 1986 concurred that ‘British paediatricians, family practitioners and child psychiatrists are far less ready than their colleagues in the USA to diagnose and treat … hyperactivity’, but also cautioned that ‘severe and pervasive hyperactivity is a risk factor and can handicap social development’ and that ‘British medicine and education will need to make its modification a higher priority’.^57^ London psychiatrist Eric Taylor similarly suggested that both British educators and physicians should recognise hyperactivity more readily, although he also suspected that it was over-diagnosed in the USA.^58^ In particular, he believed British psychiatrists should begin distinguishing hyperactivity from conduct disorder, a common British diagnosis.^59^

As the 1990s dawned, North American approaches to hyperactivity were increasingly finding favour.^60^ Many British psychiatrists argued that the differential rates of hyperactivity diagnoses in the UK and the USA were not a question of cultural difference, as some suspected, but were due to the diagnostic criteria they were using. The *DSM* criteria used by North American physicians was less stringent than that of the WHO’s International Classification of Disease, which was preferred in the UK. According to the National Institute for Health and Clinical Excellence in England and Wales (NICE), when ICD was used, only 1–2 per cent of children would be diagnosed as hyperactive; when *DSM-IV* was used the rate was 3–9 per cent. Preferring the North American criteria, Eric Taylor and psychiatrist Peter Hill published a protocol in 2001 for dealing with ADHD which was based on *DSM-IV* criteria, and, using these, subsequently estimated similar British rates to those in the USA.^61^ Given the shift in diagnostic criteria, in addition to greater public awareness, British rates of diagnosis increased considerably during the 2000s.^62^ Similarly, British researchers also began embracing the notion that ADHD was rooted in genetics, rather than environmental or social factors, embarking upon twin studies to demonstrate their theories.^63^ Finally, British psychiatrists began prescribing stimulants at a much higher rate throughout the 1990s and 2000s, suggesting that North American approaches to hyperactivity had finally infiltrated the UK.

But even as British rates of ADHD increased and stimulants became more common, significant differences remained. Although official bodies such as NICE have affirmed the validity of ADHD as a medical condition, they have also recognised that opposing positions are justifiable. NICE has also been less confident about ADHD’s causes and treatment, stating recently that: ‘The diagnosis of ADHD does not imply a medical or neurological cause … . The aetiology of ADHD involves the interplay of multiple genetic and environmental factors’.^64^ Listed alongside genetics were environmental influences, such as exposure to lead, tobacco and alcohol, diet and psychosocial factors. Stimulants were certainly mentioned in selected cases, but also important were educational, behavioural, psychological, parenting and dietary interventions.^65^

All of this has amounted to the UK adopting a less rigid and more open-ended approach to ADHD. As president of the Royal College of Psychiatrists recently said in an article in the *Guardian*, ‘there is no single solution to mental disorder, nor single approach to helping patients’.^66^ Although numerous physicians espouse American-style biomedical theories of hyperactivity, many others are sceptical, opting for a more pluralistic and multifaceted view. Despite pressure to Americanise, British understandings and experiences of ADHD have been informed by a particularly British approach to psychiatry and childhood mental illness.

## Nordic Variations

When the history of ADHD in non-Anglo countries is examined, the picture becomes even more complex.^67^ The Nordic countries, for instance, bear plenty of historical, cultural, linguistic and political similarities, often being lumped together as models of social democracy, healthy living and educational achievement. Many of them also have a history of child guidance movements that were inspired by what was happening in the USA and the UK.^68^ But in terms of ADHD, their approaches have varied enormously. The use of ADHD drugs provides a compelling example of this, where children in Iceland (now the largest per capita user of Ritalin, with even higher rates than the USA) are over ten times more likely to be prescribed drugs than those in Finland, where ADHD is seen as merely an ‘everyday educational challenge’, rather than a pathology.^69^

Sweden’s ADHD history certainly demonstrates how national circumstances have influenced how the debates surrounding the disorder have evolved. During the late 1960s, one might have thought the concept of the disorder, along with the drugs prescribed to treat it would have been unwelcome in Sweden, due largely to fears about amphetamine abuse. In fact the Swedish government decided to ban Ritalin in 1968, a move widely reported in the American media. Many Swedish physicians were supportive, believing that the benefits of the drug did not outweigh the risks of abuse, and felt resentful that American medical journals continued to advertise such dangerous products.^70^ Moreover, the general approach to child psychiatry in Sweden between 1945 and 1985, as historians Karin Zetterqvist Nelson and Bengt Sandin have argued, was initially psychoanalytical, then becoming more psychodynamic during the 1970s.^71^ During the 1940s and 1950s, the prevention of child mental health disorders by addressing social conditions and educating both professionals and parents was paramount, reflecting the political ambitions of both politicians and experts, and the vision of the Swedish welfare state.^72^ Although this preventive ethos remained during the 1970s, treatment became more of a priority, but with a focus on psychological methods, rather than psychopharmacology.

By the 1980s, however, Swedish physicians and the Swedish National Board of Health began to warm to the concept of hyperactivity and biological psychiatry. Part of this was due to external influences, and the fact that Swedish health and educational authorities began screening young children for behavioural problems, but equally important was the role of child psychiatrist Christopher Gillberg. Much as Eric Taylor would carry the flag for ADHD in the UK, Gillberg was Sweden’s most prominent ADHD advocate, also writing prolifically about autism spectrum disorders (the ADHD world is also small—Taylor gave the annual lecture at the Gillberg Neuropsychiatry Centre in 2012). During the 1970s, Swedish interest in hyperactivity was limited, but once Gillberg began to lead the charge in the early 1980s, awareness of the disorder in Sweden mushroomed in parallel to his prodigious academic output.^73^

But Gillberg’s influence on the history of ADHD in Sweden did not end there. In 2002, paediatrician Leif Elinder and sociologist Eva Kärvfe, both based at the University of Lund, began demanding access to the files from one of Gillberg’s studies, a a longitudinal study of children with DAMP (Deficits in Attention, Motor-control and Perception—a Nordic variation on hyperactivity), which began in 1977. According to journalist Jonathan Gornall, the Lund academics’ interest in Gillberg’s trial began in 1996, when Gillberg and Elinder, childhood acquaintances, met at a summer party.^74^ Elinder, who was sceptical of ADHD, wanted to discuss the issue with Gillberg, but the latter demurred. The following March, Gillberg wrote a newspaper article which claimed that 10 per cent of Swedish children had a neuropsychiatric disorder.^75^ When Elinder responded in the *Swedish Medical Journal*, his sociologist colleague Eva Kärvfe, a mother of five children herself, got involved, writing a book that questioned the validity of DAMP.^76^

Gillberg was outraged and accused Elinder and Kärvfe of basing their arguments on documents supplied by Scientology (which was opposed to psychopharmacology, as anyone who has followed the career of Tom Cruise would know). Elinder and Kärvfe, in turn, accused Gillberg of fraudulently manipulating the data for his DAMP study and demanded access his data. Gillberg refused, protesting that the material was confidential, and court action ensued, with the court ruling in 2003 that Elinder and Kärvfe were entitled to view the data. But before they could do so, Gillberg had the files shredded. Although Gillberg claimed that he was merely following the confidentiality agreement signed by the participants, the courts disagreed, resulting in fines and conditional sentences for him and his collaborators. By 2010, the case had reached the European Court of Human Rights, which ruled against Gillberg, claiming that the public had a right to know how the research had been conducted.^77^

The episode divided the Swedish scientific community, with some accusing Gillberg of being an agent for the pharmaceutical industry and others claiming Elinder and Kärvfe of having connections to Scientology. More broadly, however, the vitriolic way in which the dispute was conducted will likely contribute to widening the gulf of opinion about hyperactivity in Sweden. Although recent Swedish research, including a study on the effect of ADHD medications on criminals and reoffending rates, might suggest that proponents of the disorder in Sweden are beginning to overcome the damage done by the Gillberg affair, the ultimate effect of the episode remains to be seen.^78^ From a historical point of view, however, Gillberg’s topsy-turvy tale is a timely reminder that, while seeking out bottom-up explanations for the emergence of phenomena such as ADHD is essential, the impact of one individual acting from on high should not be dismissed.

Sweden might have experienced the most scandalous ADHD history in Scandinavia, but more banal factors have also influenced its acceptance or rejection in the other Nordic countries. A recent study on the remarkable variation in the use of ADHD drugs in Nordic countries, for instance, suggested that the availability of ADHD drugs and non-pharmacological treatments, training of mental health practitioners, and clinical practices all contributed to these differences.^79^ Moreover, such rates varied considerably within countries, as they do in the USA. In a recent Norwegian study, for instance, rates of ADHD ranged from 1.1 per cent to 3.5 per cent.^80^ While similar discrepancies were found for autism spectrum disorders, there was little variation for rates of epilepsy and cerebral palsy. Strangely, the counties with the lowest and highest rates for ADHD diagnosis respectively were Vest Agder and Aust Agder, which border one another in southern Norway; in contrast, these two counties had the lowest diagnostic levels for autism spectrum disorders. One explanation for the discrepancies was that provision of Norwegian psychiatric and paediatric services were geographically separated, possibly resulting in a lack of cooperation and coordination with respect to diagnostic practices. Although the authors assumed that the differences could ‘hardly be explained by way of underlying variations in prevalence’, with anomalies in regional diagnostic practices and the decentralisation of Norwegian child health services being blamed instead, the inverse might also be true.^81^ In other words, a centralised, rigid diagnostic regimen across Norway might result in more standardised rates, glossing over the inescapable fact that children’s behaviour—and misbehaviour—is highly subjective, no matter what criteria are applied or rating scales are used. Having said this, recent Danish research indicates considerable variation of ADHD diagnoses despite the provision of free health care.^82^ While the authors speculated that the lower rates of diagnosis in rural areas may have been due to inaccessibility of diagnostic services, it is also possible that various aspects of rural life (more exposure to nature, more exercise, less exposure to pollutants) make ADHD behaviours either less prevalent or less problematic.^83^

More research is undoubtedly needed to explore further how ADHD has been variously perceived in the Nordic countries. While some might suggest that these differences are simply due to abnormalities in diagnostic procedures and misunderstandings about treatment, this is most likely only part of the story. One indication that the picture is somewhat more complex can be found in the ADHD research conducted in Nordic countries, which has long examined the role of numerous social factors in contributing to it and other psychiatric disorders.^84^ Such a focus on the environment hearkens back to the history of child guidance in Scandinavia and, as was the case in Sweden, the connection between mental health policy and the broader social democratic project.^85^ Likewise, the recent Danish documentary *Four Letters Apart—Children in the Age of ADHD* (2013), directed by Norwegian Erlend Mo, not only hints that hyperactive behaviour can be exacerbated by a host of triggers, but also demonstrates the value of non-pharmaceutical therapy and meticulous educational interventions.^86^ Whether a child’s behaviour had been affected by maternal smoking, family breakdown, stress, placental size, parenting style, history of head injuries or any number of other factors, Nordic ADHD research demonstrates the need for more nuanced approaches and solutions.

## Socioeconomic Status and ADHD in India

Similar pluralistic thinking has jostled with aggressive attempts to market ADHD globally as a universal and essential disorder. The history of psychiatry in the colonies of the British Empire provides a useful comparison for the spread of mental disorder of American origin today. Pioneered by Waltraud Ernst, Jonathan Sadowsky and others, the history of colonial psychiatry demonstrates how it proved untenable to extend British concepts of mental health and illness to colonial contexts.^87^ As James H. Mills describes in his history of ‘native only’ asylums in British India, the Indians staffing and resident in such asylums were able to contest and confront the designs of the British.^88^ India similarly provides an intriguing example of how the concept of ADHD—and the pharmacotherapy associated with it—has both been readily adopted and heartily challenged, not only by physicians and educators, but also, and more importantly, by parents. The case of ADHD in India provides further proof that understandings and acceptance of ADHD have been shaped by social factors, in particular, by issues of class and socioeconomics.

A superficial overview of ADHD epidemiology in India might suggest that the second most populous country on the planet was rather sluggish in jumping on the ADHD bandwagon.^89^ Although one article from the late 1960s on the effectiveness of jatamansone (a drug derived from the Jatamansi root, well-known in Ayurvedic medicine for its psychoactive properties) in treating hyperactive behaviours serves as an intriguing outlier, it is difficult to find many others until the late 1980s.^90^ When studies did begin to emerge, charting the rates of the disorder in small school, hospital or outpatient populations, researchers were keen to explore whether other factors, including perinatal difficulties, family breakdown and socioeconomic status were correlated with it.^91^ Such curiosity might have indicated a certain willingness to consider multiple explanations for childhood behavioural problems, but, by the 2000s, articles were increasingly reflecting *DSM*-influenced conceptualisations of ADHD.^92^

It is no surprise that ADHD would come to India, pushed by pharmaceutical companies and pulled by Indian physicians eager to promote western medicine. But there has been another pull factor, one that has often been unappreciated in the debates about ADHD, specifically, the willingness of parents to accept an ADHD diagnosis in the hopes that it will help their children to live up to their potential. The reasons why parents have rejected, tolerated or even embraced an ADHD diagnosis are understandably complex. Such is the case in India, as in every country in the world, but here, again, local circumstances provide some explanations. According to medical anthropologist Stefan Ecks, while most Indian parents are quite reluctant to agree to ADHD drugs for their children, this is not always the case.^93^ In some aspiring middle-class neighbourhoods in Kolkata the situation is quite different, with parents seeing ADHD and the drugs that accompany it as something that can give their children an edge, particularly in the exams that serve as the gateway to prestigious universities. Given that Indian students are expected to study more and at an earlier age than in most countries, this acceptance of ADHD and drug treatment contributes to an already febrile and highly competitive educational environment. The stigma normally associated with mental disorder in Indian society is supplanted by parents’ desire to see their children get an edge in school and attain career success. Such ambitions are no different than in other parts of the world, but in lower-middle-class Kolkata, where academic achievement can be so transcendent, they are more keenly felt.

Elsewhere, however, drug treatments have been viewed suspiciously. In one study, 20 out of the 24 Indian subjects refused to adhere to their prescriptions for one month. The reasons provided for this ‘unexpected’ recalcitrance included side effects, inefficacy, fears that the drugs would lead to addiction, cost, the opposition of other family members or physicians, the ‘careless attitude of caregivers’ and the refusal of children to take the drug.^94^ All this was despite the investigators providing parents with fulsome information about ADHD and the benefits of drug treatment. Such findings were not unique; in another study, only 11 per cent of patients were still on their prescribed ADHD medications after a year.^95^

Many of the explanations cited for non-adherence centred on concerns about the drug itself or lack of understanding about the disorder (particularly complaints about children not grasping its apparent significance). But parents in other studies have presented more fundamental arguments to counter biomedical explanations for their children’s behaviour. For example, a survey of Indian parents from a lower socioeconomic grouping in Goa demonstrated greater willingness to consider psychological, educational, social or domestic explanations, including their own parenting techniques. Instead of opting for drugs, these parents tended to rely on educational or religious interventions. The researchers concluded that, regardless of the marketing strategies of pharmaceutical companies, ‘locally acceptable illness models’ needed to be employed in order to help parents of children with behavioural problems in developing countries.^96^

Other researchers have similarly focussed on even more basic factors believed to affect child behaviour. Environmental and nutritional explanations for hyperactivity have long been highly controversial, with the food additive-free Feingold diet being the most infamous, yet enduring, example.^97^ The link between industrial pollutants, such as lead and polychlorinated biphenyl (PCB), and hyperactivity, however, has quietly been acknowledged since the 1970s, when more general alarms about such chemicals were raised.^98^ Some have even hypothesised that removal of lead from gasoline in the USA, which began in 1973 and was completed in 1995, has contributed to the drop in rates of violent crime.^99^ Since such pollutants remain problematic in India, researchers have sensibly begun to explore whether children diagnosed with ADHD are actually victims of toxic exposure. Although leaded gasoline was phased out of India in 2001, resulting in drastic reductions in blood lead levels in children in cities such as Mumbai, levels are still high in many areas, especially those that are deprived. One study, which focused on Chennai, found that children from lower socioeconomic backgrounds had higher blood lead levels than their better-off peers and, correspondingly, tended to present more psychiatric problems, including ADHD-like behaviours, anxiety and detachment.^100^ The authors argued that such findings had public health implications for an industrialising country, such as India, with a young population that continues to be exposed to lead. Given that ADHD has also been associated with other factors that disproportionately affect the developing world, basic malnutrition being the most obvious example, it could well be that achieving higher nutritional and environmental standards in places like India will be far more effective in promoting mental health than adopting western psychiatric tropes and pharmaceutical cures.

## Raising Children in China

As with the history of psychiatry in the British empire, the history of Chinese psychiatry has attracted increased interest recently, as Howard Chiang’s edited collection *Psychiatry in Chinese History* attests.^101^ Chinese psychiatry, much like psychiatry in communist Europe, has been shaped by deep-rooted cultural and historical factors, as well as the prevailing political climate.^102^ In general, Chinese psychiatry has had a history of defying western psychiatric concepts, but this appears to be changing. In Chiang’s volume, for instance, Hsuan-Ying Huang describes how psychotherapy, rejected during Maoist China as bourgeois nonsense, has become quite popular amongst the wealthy of Beijing and Shanghai.^103^ ADHD also provides an example of a western, and specifically American, concept that found fertile soil in China.

Unlike in India, Chinese psychiatrists have recognised hyperactivity as being prevalent in the childhood population for decades.^104^ According to Robert J. Simmons, a Canadian child psychiatrist who was hosted by China’s Ministry of Health in 1980, in order to understand why this was the case, it was imperative to understand how children are perceived in China. It was obvious to Simmons that children were ‘revered’ in China, with the one child policy strengthening, rather than diminishing, their ‘special place in Chinese society’.^105^ Concurrently, Chinese children were also ‘well disciplined as a result of the traditional Chinese authoritarian family structure … and because of the discipline that is demanded in institutions’.^106^ Such strictness has been cited elsewhere as an explanation for why Chinese-American children rate lower for hyperactivity than their peers.^107^ In public places, such as parks, children were shy, but not fearful, able to enjoy themselves, but careful not to disturb adults. The existence of strong, multi-generational family units also meant that many emotional problems presented by children were solved by elders in the family, so much so that, if family members were unable to do so, they ran the risk of losing face.

One emerging exception to this tendency, according to Simmons, was hyperactivity, which was found to be present in children at roughly the same rates as in the USA(8–10 per cent). A large number of other studies emerged during the late 1980s and 1990s charting Chinese hyperactivity rates and comparing them to the USA, something fairly unusual for a specific disorder in China. Although amphetamines were used to treat such children, traditional herbal stimulants were also used, and most parents and teachers expected that some form of behavioural intervention also be made, rather than a complete reliance on pharmaceuticals.

While the rates tended to be similar, a common explanation for any differences was variation in child-rearing practices, with Chinese families not only being found to be stricter, but also more stable, boasting multiple parenting figures and allowing children to remain dependent on their elders for a longer period of time.^108^ Why were Chinese families willing to accept the ADHD diagnosis? According to Nanjing psychiatrist Kuo-Tai Tao, diagnoses surged in the mid-1980s following the disorder’s portrayal in a popular magazine, with many supposing that it reflected the ‘overconcern of parents’ regarding their ‘only child’ and their academic performance.^109^ Recognising that such behaviours threaten scholastic achievement and were socially unacceptable, many parents opted for Ritalin, whicht teachers referred to as the ‘be wise drug’.^110^ Others, apprehensive about western drugs, preferred traditional Chinese medicine and, still others, admitted that the ‘vicious cycle of pressures’ they placed on their children were liable to make their disruptive behaviours worse.^111^ Similarly, and much like India and Scandinavia, Chinese researchers have also been keen to explore the correlation of ADHD with external factors, ranging from parents’ physical health and education to marital problems and complications in pregnancy.^112^

In sum, China provides an interesting case for the take up of ADHD, one that bears the hallmarks of a complex society that has been shaped by ancient traditions, 65 years of communism and selective flirtations with the west. Many of the factors involved in the emergence of the disorder in the USA during the 1950s have also been present in China, helping to account for why it was accepted there long before it was in other countries.^113^ These include a ‘filiarchal’, or child-centred, society, excessive concern about educational achievement and personal discipline, and a state keen to compete at the highest level on the world stage in terms of scientific, economic and military advancement.^114^ In contrast, Chinese parents’ distrust of ADHD drugs and their preference for traditional remedies reflects attitudes about western and Chinese medicine more generally. Parental willingness to accept blame for their children’s behaviour—again, not a feature of the emergence of hyperactivity in the USA—also makes some sense in an authoritarian society where basic family decisions, such as how many children a couple should have, have been taken out of the hands of parents. Although acceptance of ADHD in China suggest that childhood there have become ‘McDonaldized’, a closer look provides some evidence that, as in other countries, the picture is not quite so clear.

## Conclusion

Worldwide, interest in ADHD continues apace. A recent study of global medical literature between 1980 and 2005 confirmed that not only had articles about ADHD grown exponentially during the period, but also that no saturation point was in sight.^115^ While there is a great deal of homogeneity in this vast body of research (the most common topic has been methylphenidate or Ritalin), the histories of ADHD in individual nations reveals a very different picture. Rather than ADHD being an essential and universal disorder, hermetically encapsulated in a *DSM* diagnosis and present in 5.29 per cent of the world’s children, these histories, only sketched out here, demonstrate that it has been a much more flexible, mutable phenomenon, a notion that has been rejected as often as it has been accepted. And, as in every history, there has always been a good story to explain why this has been the case.^116^ Historians have a vital role to play in investigating these local and national histories and, in so doing, contesting the presentation of ADHD as a global disorder that is understood synonymously worldwide. Such histories will contribute enormously not only to the history of ADHD and related disorders, but can also inform ongoing debates about ADHD, its legitimacy and what it says about different societies and their conceptualisations of childhood.

Given such a collage of different stories, then, is it possible to glean any more general lessons from ADHD’s local and national histories? One issue that emerges, which should also spur future historical research, involves the role of the child in debates about ADHD. Too often historical research on childhood disorders, such as ADHD, fails to keep the child central to the story.^117^ Equally, this has been the case in contemporary discussions of ADHD. On the one hand, ADHD has been portrayed as a powerful explanation for why children—and adults—are the way they are, why they make mistakes, struggle and fail to live up to their potential. But, on the other hand, it is important to think about who decides about what constitutes a mistake, why someone is really struggling, and how to determine an individual’s potential. Too often, and in too many places, children have been seen as the means to the end of a more competitive, prosperous, powerful society, rather than being seen as ends in themselves, as idiosyncratic individuals who should be given the opportunity to flourish unencumbered from the demands of adults who have proven generation after generation not to have many answers to society’s problems. Perhaps, instead of expecting all children, the world over, to conform to specific, *DSM*-determined criteria of behaviour, learning and development, we should do the opposite, and encourage creativity, courage and flexibility in our children, and in ourselves.

## Footnotes

1 American Psychiatric Association, *Diagnostic and Statistical Manual of Mental Disorders* (Washington, DC: American Psychiatric Association, 2013).

2 M. Smith, *Hyperactive: The Controversial History of ADHD* (London: Reaktion, 2012), 75–7.

3 H. Kutchins, and S. A. Kirk, *Making Us Crazy: DSM, the Psychiatric Bible and the Creation of Psychiatric Disorders* (New York: Free Press, 1999).

4 E. Watters, *Crazy Like Us: The Globalization of the American Psyche* (New York: Free Press, 2010).

5 P. M. Doraiswamy, ‘Are We Homogenizing the Global View of a Normal Mind?’ *Edge*, 2013, <https://www.edge.org/response-detail/23855>, accessed 6 May 2016.

6 M. W. Laufer, E. Denhoff and G. Solomons, ‘Hyperkinetic Impulse Disorder in Children’s Behavior Problems’, *Psychosomatic Medicine*, 1957, 19, 45.

7 G. Polanczck, M. Silva de Lima, B. L. Horta, J.Biederman and L. A. Rohde, ‘The Worldwide Prevalence of ADHD: A Systematic and Metaregression Analysis’, *American Journal of Psychiatry*, 2007, 164, 942–8.

8 T. E. Moffit and M. Melchior, ‘Why Does the Worldwide Prevalence of Childhood Attention Deficit Hyperactivity Disorder Matter’, *American Journal of Psychiatry*, 2007, 64, 856.

9 D. P. Béhague, ‘Psychiatry and Politics in Pelotas, Brazil: The Equivocal Quality of Conduct Disorder and Related Diagnoses’, *Medical Anthropology Quarterly*, 2009, 23, 45–82.

10 S. V. Faraone, J. Sergeant, C. Gillberg and J. Biederman, ‘The Worldwide Prevalence of ADHD: Is it an American Condition?’ *World Psychiatry*, 2003, 2, 104–13.

11 Centers for Disease Control and Prevention, ‘Increasing Prevalence of Parent-Reported Attention-Deficit/Hyperactivity Disorder Among Children—United States, 2003 and 2007’, *Morbidity and Mortality Weekly Report*, 2010, 59, 1439–43.

12 S. Timimi, ‘The McDonaldization of Childhood: Children’s Mental Health in Neo-Liberal Cultures’, *Transcultural Psychiatry*, 2010, 47, 686–706.

13 There are numerous recent histories that have begun to use local analyses as a way of developing a more sophisticated understanding of health, illness and medicine. See E. Dyck and C. Fletcher, (eds), *Locating Health: Historical and Anthropological Investigations of Place and Health* (London: Pickering and Chatto, 2010); J. S. Stark, *The Making of Modern Anthrax, 1875–1920: Uniting Local, National and Global Histories of Disease* (London: Pickering and Chatto, 2013); L. Monnais and H. Cook, (eds), *Global Movements, Local Concerns: Medicine and Health in Southeast Asia* (Singapore: Singapore University Press, 2014).

14 Smith, *Hyperactive*.

15 Here I am attempting in a modest way to answer Ilana Löwy’s call for more comparative social histories of medicine. I. Löwy, ‘The Social History of Medicine: Beyond the Local’, *Social History of Medicine*, 2007, 20, 465–81.

16 There have also, of course, been variations *within* countries as well. I have indicated some of these national variations, but most of my analysis is at the national level for the sake of comparison.

17 On the possible promotion by psychiatrists and pharmaceutical companies, see S. Timimi and B. Maitra, ‘ADHD and Globalization’ in Sami Timimi and Jonathan Leo, eds, *Rethinking ADHD: From Brain to Culture* (Basingstoke: Palgrave Macmillan, 2009); Timimi, ‘McDonaldization of Childhood’.

18 P. E. Trudeau, ‘Speech to the Press Club’, 25 March 1969, <http://www.cbc.ca/player/play/1797537698>, accessed 22 August 2014.

19 E. Kromer, ‘Sleeping with the Elephant’, *The McGill Reporter* 21 November 2002, <http://www.mcgill.ca/reporter/35/06/reinhardt/>, accessed 22 August 2014.

20 P. Sullivan, ‘Privatization if Necessary, Not Necessarily Privatization: CMA’, 19 August 2005, accessed 22 August 2014. For an excellent series of articles on the history of Medicare in Canada, see the special issue of the *Canadian Bulletin of Medical History* (Volume 26, Number 2, 2009) edited by Gregory P. Marchildon.

21 J. Duffin and L. A. Falk, ‘Sigerist in Saskatchewan: The Quest for Balance in Social and Technical Medicine’, *Bulletin of the History of Medicine*, 1996, 70, 658–83.

22 M. W. Laufer and E. Denhoff, ‘Hyperkinetic Behavior Syndrome in Children’, *Journal of Pediatrics*, 1957, 50, 463–74; M. W. Laufer, E. Denhoff and G. Solomons, ‘Hyperkinetic Impulse Disorder in Children’s Behavior Problems’, *Psychosomatic Medicine*, 1957, 19, 38–49. For the later debate, see J. S. Werry, Gabrielle Weiss and Virginia Douglas, ‘Studies on the Hyperactive Child—I. Some Preliminary Findings’, *Canadian Psychiatric Association Journal*, 1964, 9, 120–30.

23 J. Mackay, ‘The Psychiatric Problems of the Teenager’, *Canadian Family Physician*, 1968, 14, 21–6.

24 P. S. Stephenson, ‘Drugs in Child Psychiatry’, *Canadian Family Physician*, 1969, 15, 30–2.

25 McGill University was the site of a great deal of highly influential medical research during the mid-twentieth century, including the pioneering work of stress theorist Hans Selye (1907–1982) and neurosurgeon Wilder Penfield (1891–1976). See D. Gavrus, ‘Men of Dreams and Men of Action: Neurologists, Neurosurgeons, and the Performance of Professional Identity, 1920–1950’, *Bulletin of the History of Medicine* 2011, 85, 57–92; M. Jackson, *The Age of Stress: Science and the Search for Stability* (Oxford: Oxford University Press, 2013); C. Canning; G. Weisz, A. Tone and A. Cambrosio, ‘Medical Genetics at McGill: The History of a Pioneering Research Group’, *Canadian Bulletin of Medical History*, 2013, 30, 31–54.

26 The Montreal study is reported in J. S. Werry, G. Weiss, V. Douglas and J. Martin, ‘Studies on the Hyperactive Child III: The Effects of Chlorpromazine upon Behavior and Learning Ability’, *Journal of the American Academy of Child Psychiatry*, 1966, 5, 292–312 and the follow-up study can be found in G. Weiss, E. Kruger, U. Danielson and M. Elman, ‘Effect of Long-term Treatment of Hyperactive Children with Methylphenidate’, *Canadian Medical Association Journal*, 1975, 112, 159–65; L. Hechtman, G. Weiss, J. Finkelstein, A. Werner and R. Benn, ‘Hyperactives as Young Adults: Preliminary Report’, *Canadian Medical Association Journal*, 1976, 115, 625–30; L. Hechtman, G. Weiss and K. Metrakos, ‘Hyperactive Individuals as Young Adults: Current and Longitudinal Electroencephalographic Evaluation and its Relation to Outcome’, *Canadian Medical Association Journal*, 1978, 118, 919–23; G. Weiss, L. Hechtman, T. Perlman, J. Hopkins and A. Wener, ‘Hyperactives as Young Adults: A Controlled Prospective Follow-Up of 75 Children’, *Archives of General Psychiatry*, 1979, 36, 675–81; L. Hechtman and G. Weiss, ‘Long-term Outcome of Hyperactive Children’, *American Journal of Orthopsychiatry*, 1983, 53, 532–41; B. Greenfield, L. Hechtman and G. Weiss, ‘Two Subgroups of Hyperactives as Adults: Correlations of Outcome’, *Canadian Journal of Psychiatry*, 1986, 31, 505–8.

27 N. Carrey, ‘Interview with Dr. Gabrielle “Gaby” Weiss’, *Journal of the Canadian Academy of Child and Adolescent Psychiatry*, 2004, 18, 340–3.

28 G. Weiss, L. Hechtman and T. Perlman, ‘Hyperactives as Young Adults: School, Employer, and Self-Rating Scales Obtained during Ten-Year Follow-Up Evaluation’, *American Journal of Orthopsychiatry*, 1978, 48, 438–45.

29 K. K. Minde, and N. J. Cohen, ‘Hyperactive Children in Canada and Uganda: A Comparative Evaluation’, *Journal of the American Academy of Child Psychiatry*, 1978, 17, 476–87.

30 K. K. Minde, ‘The Hyperactive Child’, *Canadian Medical Association Journal*, 1975, 112, 130–1.

31 N. J. Cohen, and K. Minde, ‘The “Hyperactive Syndrome” in Kindergarten: Comparison of Children with Pervasive and Situational Symptoms’, *Journal of Child Psychology and Psychiatry*, 1983, 24, 443–55.

32 D. H. Sykes, V. I. Douglas, G. Weiss and K. K. Minde, ‘Attention in Hyperactive Children and the Effect of Methylphenidate (Ritalin)’, *Journal of Child Psychology and Psychiatry*, 1971, 12, 129–39.

33 D. H. Sykes, V. I. Douglas and G. Morgenstern, ‘The Effect of Methylphenidate (Ritalin) on Sustained Attention in Hyperactive Children’, *Psychopharmacologia*, 1972, 25, 262–74; D. H. Sykes, V. I. Douglas and G. Morgenstern, ‘Sustained Attention in Hyperactive Children’, *Journal of Child Psychology and Psychiatry*, 1973, 14, 213–20; V. I. Douglas, ‘Stop, Look and Listen: The Problem of Sustained Attention and Impulse Control in Hyperactive and Normal Children’, *Canadian Journal of Behavioural Science*, 1972, 4, 259–82.

34 J. E. Goggin, ‘Sex Difference in the Activity Level of Preschool Children as a Possible Precursor of Hyperactivity’, *Journal of Genetic Psychology*, 1975, 127, 75–81; A. James and E. Taylor, ‘Sex Differences in the Hyperkinetic Syndrome of Childhood’, *Journal of Child Psychology and Psychiatry*, 1990, 31, 437–46.

35 P. O. Quinn, ‘Treating Adolescent Girls and Women with ADHD: Gender-Specific Issues’, *Journal of Clinical Psychology*, 2005, 61, 579–87.

36 R. Holland, ‘Hyperactivity in Children’, *Canadian Medical Association Journal*, 1989, 140, 896–97.

37 Canadian Paediatric Society, ‘Use of Methylphenidate for Attention Deficit Hyperactivity Disorder’, *Canadian Medical Association Journal*, 1990, 142, 817–18.

38 P. Graham and M. Rutter, ‘Organic Brain Syndrome: Child Psychiatric Disorder’, *British Medical Journal (BMJ)*, 1968, 3, 695–700.

39 J. L. Rapoport, I. T. Lott, D. F. Alexander and A. U. Abramson, ‘Urinary Noradrenaline and Playroom Behaviour in Hyperactive Children’, *Lancet*, 1970, 296, 1141; M. A. Stewart, ‘Urinary Noradrenaline and Playroom Behaviour in Hyperactive Children’, *Lancet*, 1971, 297, 140.

40 C. Ounsted, ‘The Hyperkinetic Syndrome in Epileptic Children’, *Lancet*, 1955, 269, 303–11; T. T. S. Ingram, ‘A Characteristic Form of Overactive Behaviour in Brain Damaged Children’, *Journal of Mental Science*, 1956, 102, 550–8; F. E. Kratter, ‘The Physiognomic, Psychometric, Behavioural and Neurological Aspects of Phenylketonuria’, *Journal of Mental Science*, 1959, 105, 421–7.

41 M. Knipe, ‘“Concentration Drug” Used in Schools’, *The Times*, 1 July 1970; M. Vaughan, ‘Schoolchildren “Put On Drugs Because Class Behaviour Does Not Fit”’ *The Times*, 1 December 1977.

42 D. V. M. Bishop, ‘Which Neurodevelopmental Disorders Get Researched and Why?’ *PloS One*, 2010, 5, e15112.

43 J. Stewart, *Child Guidance in Britain, 1918–1955: The Age of Dangerous Childhood* (London: Pickering and Chatto, 2013), 4. For the history of child guidance in the USA, see K. W. Jones, *Taming the Troublesome Child: American Families, Child Guidance, and the Limits of Psychiatric Authority* (Cambridge, MA: Harvard University Press, 1999).

44 Stewart, *Child Guidance*, 1–4. The history of child guidance in other parts of Europe similarly indicate how local factors had a major bearing on the take-up of American notions of child guidance. See T. Feeney, ‘Church, State, and Family: The Advent of Independent Child Guidance Clinics in Independent Ireland’, *Social History of Medicine*, 2012, 25, 848–62; D. Kritsotaki, ‘Mental Hygiene and Child Guidance in Post-War Greece: The Case of the Centre for Mental Health and Research, 1956–1970’, *Social History of Medicine*, 2014, 27, 751–67; A. M. Filipe, ‘The Rise of Child Psychiatry in Portugal: An Intimate Social and Political History, 1915–1959’, *Social History of Medicine*, 2014, 27, 326–48.

45 S. Box, ‘Preface’, in Peter Schrag and Diane Divoky, *The Myth of the Hyperactive Child: And Other Means of Child Control* (New York: Penguin, 1981 [1975]), 17, 23.

46 L. Hersov quoted in R. Cullen, ‘Should Naughty Children Be Drugged?’ *The Times* (15 September 1981).

47 M. Rutter, ‘Brain Damage Syndromes in Childhood: Concepts and Findings’, *Journal of Child Psychology and Psychiatry*, 1977, 18, 18.

48 M. Rutter, J. Tizzard, W. Yule, P. Graham and T. Whitmore, ‘Research Report: Isle of Wight Studies, 1964–1974’, *Psychological Medicine*, 1976, 6, 313–32.

49 J. E. Oliver and A. H. Buchanan, ‘Generations of Maltreated Children and Multiagency Care in One Kindred’, *British Journal of Psychiatry*, 1979, 135, 289–303; N. Richman, J. E. Stevenson and P. Graham, ‘Prevalence of Behaviour Problems in 3-Year-Old Children’, *Journal of Child Psychology and Psychiatry*, 1975, 16, 285.

50 Anonymous, ‘Hyperactivity’, *Lancet*, 1978, 312, 561.

51
*Ibid*.

52 Anonymous, ‘Does Hyperactivity Matter?’ *Lancet*, 1986, 327, 73–4; Mark A. Stewart and B. H. Burne, ‘Minimal Brain Dysfunction’, *Lancet*, 1973, 302, 852.

53 Box, ‘Preface’, 24.

54 Anonymous, ‘Feingold’s Regimen for Hyperkinesis’, *Lancet*, 1979, 314, 617–18; T. Fishlock, ‘Lead in Petrol: Bad for Cars but Far Worse for Children’s Health’, *The Times*, 13 December 1979; Anonymous, ‘Food Additives and Hyperactivity’, *Lancet*, 1982, 319, 662–3; D. Wilson, ‘Petrol: Must Our Children Still Be Poisoned?’ *The Times*, 8 February 1982; S. Greaves, ‘With Added Goodness?’ *The Times*, 21 August 1985; M. Smith, *An Alternative History of Hyperactivity: Food Additives and the Feingold Diet* (New Brunswick, NJ: Rutgers University Press, 2011).

55 Smith, *Alternative History of Hyperactivity*, 157–61.

56 G. Thorley, ‘Hyperkinetic Syndrome of Childhood: Clinical Characteristics’, *British Journal of Psychiatry*, 1984, 144, 16–24.

57 Anonymous, ‘Does Hyperactivity Matter?’

58 E. A. Taylor, ‘Childhood Hyperactivity’, *British Journal of Psychiatry*, 1986, 149, 562–73.

59 E. A. Taylor, R. Schachar, G. Thorley and M. Wieselberg, ‘Conduct Disorder and Hyperactivity: I. Separation of Hyperactivity and Antisocial Conduct in British Child Psychiatric Patients’, *British Journal of Psychiatry*, 1986, 149, 760–7.

60 This was at roughly the same time as American approaches to healthcare were influencing the Thatcher government. See A. Enthoven, *Reflections on the Management of the of the National Health Service: An American Looks at Incentives to Efficiency in Health Services Management in the UK* (London: The Nuffield Provincial Hospitals Trust, 1985).

61 P. Hill and E. Taylor, ‘An Auditable Protocol for Treating Attention Deficit/Hyperactivity Disorder’, *Archives of Disease in Childhood*, 2001, 84, 404–9; T. Alloway, J. Elliott and J. Holmes, ‘The Prevalence of ADHD-Like Symptoms in a Community Sample’, *Journal of Attention Disorders*, 2010, 14, 52–6.

62 NICE, Attention Deficit Hyperactivity Disorder: Diagnosis and Management of ADHD in Children, Young People and Adults (London: NICE, 2009), <http://www.nice.org.uk/nicemedia/live/12061/42060/42060.pdf>, accessed 24 August 2014.

63 A. Thapar, R. Harrington, K. Ross and P. McGuffin, ‘Does the Definition of ADHD Affect Heritability?’ *Journal of the American Academy of Child and Adolescent Psychiatry*, 2000, 39, 1528–36.

64 NICE, Attention Deficit Hyperactivity Disorder, 28–9.

65 It should be noted, however, that the Scottish Intercollegiate Guidelines Network (SIGN) for ADHD are much more forthright in recommending the use of psychostimulants as a first course of action. Scottish Intercollegiate Guidelines Network, *Management of Attention Deficit and Hyperkinetic Disorders in Children and Young People: A National Clinical Guideline*, <http://www.sign.ac.uk/pdf/sign112.pdf>, accessed 6 May 2016.

66 S. Wessely, ‘The “Drugs v Talking” Debate Doesn’t Help Us Understand Mental Health’, *The Guardian*, 29 March 2016, <http://www.theguardian.com/commentisfree/2016/mar/29/drugs-talking-mental-health>, accessed 8 May 2016.

67 In terms of Australia and New Zealand, very broadly speaking, the history of ADHD has been somewhat of a combination of the Canadian and British experiences: early exposure to the disorder, as in Canada, but considerable interest in alternative, particularly ecological, explanations, as in the UK.

68 R. Qvarsell, ‘History of Psychiatry in Sweden’, *History of Psychiatry*, 1991, 2, 315–20; H. Hannesdóttir, ‘Child and Adolescent Psychiatry in Iceland: The State of the Art, Past, Present, and Future’, *Nordic Journal of Psychiatry*, 1993, 47, 9–13; K. Ludvigsen and Ä. A. Seip, ‘The Establishing of Norwegian Child Psychiatry: Ideas, Pioneers and Institutions’, *History of Psychiatry*, 2009, 20, 5–26; K. Ludvigsen, ‘The “psy-experts” and the Minds of Children: Transfer of Knowledge in Inter-War Norway and Sweden’ in Å Lundkvist and K. Peterson, eds, *In Experts We Trust: Knowledge, Politics and Bureaucracy in Nordic Welfare States* (Odense: University Press of Southern Denmark, 2010), 181–212; A. L. Dixon and N. H. Hansen, ‘*Fortid*, *Nutid*, *Fremtid* (Past, Present, Future): Professional Counselling in Denmark’, *Journal of Counseling and Development*, 2010, 88, 38–42; P. Pietikainen, ‘Finland’, in D. B. Baker, ed., *Oxford Handbook of the History of Psychology* (Oxford: Oxford University Press, 2012), 211–27.

69 M. Jahnukainen, ‘Different Children in Different Countries: ADHD in Canada and Finland’, in L. J. Graham, ed,, *(De)Constructing ADHD: Critical Guidance for Teachers and Teacher Educators* (New York: Peter Lang, 2009), 63; *International Narcotics Control Board, Report of the International Narcotics Control Board for 2009* (New York: United Nations, 2010).

70 E. Perman, ‘Speed in Sweden’, *New England Journal of Medicine*, 1970, 283, 760–1.

71 K. Zetterqvist Nelson and Bengt Sandin, ‘Psychodynamics in Child Psychiatry in Sweden, 1945–1985: From Political Vision to Treatment Ideology’, *History of Psychiatry*, 2014, 24, 308–25.

72
*Ibid*., 312–14.

73 For the position in the 1970s, see L. Köhler, ‘Physical Examination of Four-Year-Old Children’, *Acta Paediatrica Scandinavia*, 1973, 62, 181–92; B. Hagberg, ‘Minimal Brain Dysfunction in Children: Effects on Development and Adjustment’, *Läkartidningen*, 1975, 72, 3296–300; T. Korhonen and M. Sillanpää, ‘MBD-Like Behavior and Neuropsychological Performance’, *Acta Paedopsychiatrica*, 1975, 42, 75–87. C. Gillberg, P. Rasmussen, G. Carlström, B. Svenson and E. Waldenström, ‘Perceptual, Motor and Attentional Deficits in Six-Year-Old Children. Epidemiological Aspects’, *Journal of Child Psychology and Psychiatry*, 1982, 25, 131–44 provide a view of later developments.

74 J. Gornall, ‘Hyperactivity in Children: The Gillberg Affair’, *British Medical Journal*, 2007, 335, 370–3.

75 C. Gillberg, ‘School Ruins 120,000 Children’, *Dagens Nyheter*, 20March 1997.

76 E. Kärvfe, *Hjärnspöken—DAMP och hotet mot folkhälsan* (Stockholm: Symposion, 2000).

77 European Court of Human Rights, ‘Case of Gillberg v. Sweden’, 2 November 2010, <http://merlin.obs.coe.int/iris/2012/6/article1.en.html>, accessed 24 August 2014.

78 P. Lichtenstein, L. Haldner, J. Zetterqvist, A. Sjölander, E. Serlachius, S. Fazel, N. Lângström and H. Larsson, ‘Medication for Attention Deficit-Hyperactivity Disorder and Criminality’, *New England Journal of Medicine*, 2012, 367, 2006–14.

79 H. Zoëga, K. Furu, M. Halldórsson, P. H. Thomsen, A. Sourander and J. E. Martikainen, ‘Use of ADHD Drugs in Nordic Countries: A Population-Based Comparison Study’, *Acta Psychiatrica Scandinavia*, 2011, 123, 360–7.

80 P. Surén, I. J. Bakken, K. K. Lie, S. Schjølberg, H. Aase, T. Reichborn-Kjennerud, P. Magnus, A. Øyen, B. K. Svendsen, K. M. Aaberg, G. L. Andersen and C. Stoltenberg, ‘Differences Across Counties in the Registered Prevalence of Autism, ADHD, Epilepsy and Cerebral Palsy’, *Tidsskrift for Den norske legeforening*, 2013, 18, 1929–34.

81
*Ibid*.

82 K. B. Madsen, A. K. Ersbøll, J. Olsen, E. Parner and C. Obel, ‘Geographic Analysis of the Variation in the Incidence of ADHD in a Country with Free Access to Healthcare: A Danish Cohort Study’, *International Journal of Health Geographics*, 2015, 14, 1–13.

83 See R. Louv, *Last Child in the Woods: Saving Our Children from Nature Deficit Disorder* (Chapel Hill, NC: Algonquin Books, 2005); B. W. Wheeler, M. Whyte, W. Stahl-Timmins and M. H. Depledge, ‘Does Living by the Coast Improve Health and Wellbeing?*, Health and Place*, 2012, 18, 1198–201; M. Thomson, *Lost Freedom: The Landscape of the Child and the British Post-War Settlement* (Oxford: Oxford University Press, 2014).

84 O. Östman, ‘Patients in Child and Adolescent Psychiatric Care: Psychopathology and Background Factors’, *Acta Psychiatrica Scandinavia*, 1989, 80, 408–14.

85 Nelson and Sandin, ‘Psychodynamics in Child Psychiatry in Sweden’.

86 E. E. Mo, *Four Letters Apart—Children in the Age of ADHD* (Copenhagen, Magic Hour Films, 2013).

87 W Ernst, *Mad Tales from the Raj: The European Insane in British India* (London: Routledge, 1991); W. Ernst, ‘European Madness and Gender in Nineteenth-Century British India’, *Social History of Medicine*, 1996, 9, 357–82; J. Sadowsky, *Imperial Bedlam: Institutions of Madness in Colonial Southwest Nigeria* (Berkeley: University of California Press, 1999); J. H. Mills, *Madness, Cannabis, and Colonialism: The ‘Native Only’ Lunatic Asylums of British India, 1857–1900* (Basingstoke: Macmillan, 2000); S. Mahone and M. Vaughan, eds, *Psychiatry and Empire* (Basingstoke: Palgrave Macmillan, 2007); A. McCarthy and C. Coleborne, eds, *Migration, Ethnicity, and Mental Health: International Perspectives, 1840–2010* (Abingdon: Routledge, 2010); W. Ernst, *Colonialism and Transnational Psychiatry: The Case of an Indian Mental Hospital in British India, c. 1920–1940* (London: Anthem, 2013); J. McCulloch, *Black Skin, White Coats: Nigerian Psychiatrists, Decolonization, and the Globalization of Psychiatry* (Athens: Ohio University Press, 2013).

88 Mills, *Madness, Cannabis, and Colonialism*.

89 S. G. Crawford, ‘Specific Learning Disabilities and Attention-Deficit Hyperactivity Disorder: Under-Recognized in India’, *Indian Journal of Medical Science*, 2007, 61, 637–8.

90 P. D. Gupta and V. Virmani, ‘Clinical Trial of Jatamansone (syn. Valeranone) in Hyperkinetic Behaviour Disorders’, *Neurology India*, 1968, 16, 168–73.

91 M.S. Bhatia, V. R. Nigam, N. Bohra and S. C. Malik, ‘Attention Deficit Disorder with Hyperactivity Among Paediatric Outpatients’, *Journal of Child Psychology and Psychiatry*, 1991, 32, 297–306.

92 B. S. Suvarna and A. Kamath, ‘Prevalence of Attention Deficit Disorders in Preschool Age Children’, *Nepalese Medical College Journal*, 2009, 11, 1–4.

93 S. Ecks, *Eating Drugs: Psychopharmaceutical Pluralism in India* (New York: New York University Press, 2013), 184–5.

94 P. Sitholey, V. Agarwal and S. Chamoli, ‘A Preliminary Study of Factors Affecting Adherence to Medication in Clinic Children with Attention-Deficit/Hyperactivity Disorder’, *Indian Journal of Psychiatry*, 2011, 53, 41.

95 D. Seth, ‘A Follow-Up Study of Clinic Children and Adolescents with Attention Deficit Hyperactivity Disorder’, PhD Dissertation, Lucknow University, 2002.

96 C. E. Wilcox, R. Washburn and V. Patel, ‘Seeking Help for Attention Deficit Hyperactivity Disorder in Developing Countries: A Study of Parental Explanatory Models in Goa, India’, *Social Science and Medicine*, 2007, 64, 1600.

97 Smith, *Alternative History of Hyperactivity*.

98 P. A. Eubig, A. Aguiar and S. L. Schantz, ‘Lead and PCBs as Risk Factors for Attention Deficit/Hyperactivity Disorder’, *Environmental Health Perspectives,* 2010, 118, 1654–67.

99 K. Drum, ‘America’s Real Criminal Element: Lead’, *Mother Jones*, January/February, 2013.

100 A. Roy, D. Bellinger, H. Hu, J. Schwartz, A. S. Ettinger, R. O. Wright, M. Bouchard, K. Palaniappan and K. Balakrishnan, ‘Lead Exposure and Behavior Among Young Children in Chennai, India’, *Environmental Health Perspectives*, 2009, 117, 1607–11.

101 H. Chiang (ed.), *Psychiatry and Chinese History* (London: Pickering and Chatto, 2014).

102 On psychiatry in Communist countries generally, see M. Savelli and S. Marks, eds, *Psychiatry in Communist Europe* (Basingstoke: Palgrave Macmillan, 2015). On Chinese psychiatry more specifically, see for instance, see N. Chen, *Breathing Spaces: Qigong, Psychiatry, and Healing in China* (New York: Columbia University Press, 2003); H. Y. Wu, ‘The Moral Career of “Outmates”: Towards a History of Manufactured Mental Disorders in Post-Socialist China’, *Medical History*, 2016, 60, 87–104.

103 H. Huang, ‘The Emergence of the Psycho-Boom in Contemporary China’, in H. Chiang, ed., *Psychiatry and Chinese History* (London: Pickering and Chatto, 2014), 183.

104 J. D. Xin, H. Y. Liao, D. C. Xu and Y. F. Xia, ‘Clinical Analysis of 700 Cases with Minimal Brain Dysfunction’. *Chinese Journal of Nervous and Mental Disease*, 1981, 7, 226–30; Y. C. Shen, Y. F. Wang and X. L. Yang ‘An Epidemiological Investigation of Minimal Brain Dysfunction in Six Elementary Schools in Beijing’, *Journal of Child Psychology and Psychiatry,* 1985, 26, 777–87.

105 R. J. Simmons, ‘Observations of Child Psychiatry in China’, *Canadian Journal of Psychiatry*, 1983, 28, 125.

106
*Ibid*.

107 K. N. Yao, M. V. Solanto and E. H. Wender, ‘Prevalence of Hyperactivity Among Newly Immigrated Chinese-American Children’, *Journal of Developmental and Behavioral Pediatrics*, 1988, 9, 367–73.

108 X. Li, R., L. Y. Su, B. D. Townes and C. K. Varley, ‘Diagnosis of Attention Deficit Disorder with Hyperactivity in Chinese Boys’, *Journal of the American Academy of Child and Adolescent Psychiatry*, 1989, 28, 497–500.

109 K. Tao, ‘Hyperactivity and Attention Deficit Disorder Syndromes in China’, *Journal of the American Academy of Child and Adolescent Psychiatry*, 1992, 31, 1165.

110
*Ibid*.

111 On preferences for traditional Chinese medicine, see X. Gai, G. Lan and X. Lui, ‘A Meta-Analytic Review on Treatment Effects on Attention Deficit/Hyperactivity Disorder Children in China’, *Acta Psychologica Sinica*, 2008, 40, 1190–96. The quote is from Tao, ‘Hyperactivity’, 1166.

112 R. Xin, S. K. Chen, H. Q. Tang, S. F. Lin and B. J. McConville, ‘Behavioural Problems among Preschool Age Children in Shanghai: Analysis of 3,000 Cases’, *Canadian Journal of Psychiatry*, 1992, 37, 251–8; C. K. Wong, ‘Psychiatric Morbidity in a Chinese Primary School in Hong Kong’, *Australian and New Zealand Journal of Psychiatry*, 1992, 26, 459–66.

113 Smith, *Hyperactive*.

114 S. Mintz and S. Kellogg, *Domestic Revolutions: A Social History of Family Life* (New York: Free Press, 1989), 184–7.

115 F. López-Muñoz, C. Alamo, F. J. Quintero-Gutiérrez and P. García-García, ‘A Bibliometric of International Scientific Productivity in Attention-Deficit Hyperactivity Disorder Covering the Period 1980–2005, *European Child and Adolescent Psychiatry*, 2008, 17, 381–91.

116 S. P. Hinshaw, R. M. Scheffler, B. D. Fulton, H. Aase, T. Banaschewski, W. Cheng, P. Mattos, A. Holte, F. Levy, A. Sadeh, J. A. Sergeant, E. Taylor and M. D. Weiss, ‘International Variation in Treatment Procedures for ADHD: Social Contexts and Recent Trends’, *Psychiatric Services*, 2011, 62, 459–64.

117 There are, of course, exceptions to this. See Hannah Newton, *The Sick Child in Early Modern England, 1580–1720* (Oxford: Oxford University Press, 2012).

## Notes

Matthew Smith is Professor of Health History at the University of Strathclyde's Centre for the Social History of Health and Healthcare, which he co-directs. His books include *Another Person's Poison: A History of Food Allergy* (Columbia UP, 2015); *Hyperactive: The Controversial History of ADHD* (Reaktion, 2012) and *An Alternative History of Hyperactivity: Food Additives and the Feingold Diet* (Rutgers UP, 2011).

